# Reconstructing Macroevolutionary Patterns in Avian MHC Architecture With Genomic Data

**DOI:** 10.3389/fgene.2022.823686

**Published:** 2022-02-17

**Authors:** Ke He, Chun-hong Liang, Ying Zhu, Peter Dunn, Ayong Zhao, Piotr Minias

**Affiliations:** ^1^ College of Animal Science and Technology, College of Veterinary Medicine, Zhejiang Agriculture and Forestry University, Key Laboratory of Applied Technology on Green-Eco-Healthy Animal Husbandry of Zhejiang Province, Hangzhou, China; ^2^ Institute of Qinghai-Tibetan Plateau, Southwest Minzu University, Chengdu, China; ^3^ Behavioral and Molecular Ecology Group, Department of Biological Sciences, University of Wisconsin-Milwaukee, Milwaukee, WI, United States; ^4^ Department of Biodiversity Studies and Bioeducation, Faculty of Biology and Environmental Protection, University of Łodz, Łódź, Poland

**Keywords:** MHC architecture, MHC gene structure, avian MHC, macroevolutionary, third-generation sequencing genome

## Abstract

The Major Histocompatibility Complex (MHC) is a hyper-polymorphic genomic region, which forms a part of the vertebrate adaptive immune system and is crucial for intra- and extra-cellular pathogen recognition (MHC-I and MHC-IIA/B, respectively). Although recent advancements in high-throughput sequencing methods sparked research on the MHC in non-model species, the evolutionary history of MHC gene structure is still poorly understood in birds. Here, to explore macroevolutionary patterns in the avian MHC architecture, we retrieved contigs with antigen-presenting MHC and MHC-related genes from available genomes based on third-generation sequencing. We identified: 1) an ancestral avian MHC architecture with compact size and tight linkage between MHC-I, MHC-IIA/IIB and MHC-related genes; 2) three major patterns of MHC-IIA/IIB unit organization in different avian lineages; and 3) lineage-specific gene translocation events (e.g., separation of the antigen-processing TAP genes from the MHC-I region in passerines), and 4) the presence of a single MHC-IIA gene copy in most taxa, showing evidence of strong purifying selection (low dN/dS ratio and low number of positively selected sites). Our study reveals long-term macroevolutionary patterns in the avian MHC architecture and provides the first evidence of important transitions in the genomic arrangement of the MHC region over the last 100 million years of bird evolution.

## Introduction

The major histocompatibility complex (MHC) is a central component of the vertebrate adaptive immune system, containing antigen-presenting class I (MHC-I) and class II (MHC-II) genes, which are primarily responsible for recognition of intra- and extra-cellular pathogens (Blum et al., 2013). Both MHC-I and MHC-II antigen-presenting molecules have a two-domain peptide-binding region (coded by exon 2 and 3 of a single MHC-I gene and by exon 2 of MHC-IIA and MHC-IIB genes), which directly bind to peptides. Although both MHC-I and MHC-II molecules have heterodimeric structure, only at the MHC-II is this heterodimerization apparent within the peptide-binding region (α and β chains coded by IIA and IIB genes) (Ting and Trowsdale, 2002). The peptide-binding region is often subject to strong pathogen-driven balancing selection and retains high level of polymorphism within populations (Zeng et al., 2016; Biedrzycka et al., 2017; Whittingham et al., 2018). Nevertheless, the MHC region also contains other MHC-related genes involved in antigen processing (e.g., transporters associated with antigen processing, TAPs), which show varying degree of linkage with antigen-presenting genes (Lankat-Buttgereit and Tampë, 1999).

A combination of high-throughput sequencing technology and development of conservative primers for targeted amplification of peptide-binding regions (Alcaide et al., 2013) has sparked extensive research on the population-wide MHC polymorphism in non-model organisms (including birds) (Biedrzycka et al., 2017; Minias et al., 2019; Qin et al., 2021). However, due to pseudogenization and duplication processes at the MHC (O'Connor et al., 2019; O'Connor and Westerdahl, 2021), characterization of its genomic architecture still constitutes a major challenge. In fact, gene arrangement, haplotype inference and linkage relationships at the avian MHC have all been clearly under-researched, mostly due to technical limitations (O'Connor et al., 2019).

Among birds, the architecture of MHC was first resolved in the chicken *Gallus gallus* (Kaufman et al., 1999), in which MHC-I, MHC-IIB, and other MHC-related genes lay within a single core region of less than 100 kb in length (chromosome 16), while MHC-IIA is considered to be located roughly 5.6 cM away on the same chromosome (Kaufman et al., 1999; Salomonsen et al., 2003). Several other landfowl species (Galliformes) have been reported to retain a conserved structure of the MHC-related core region, despite gene copy number variation (2-7 MHC-I genes and 1-3 MHC-IIB genes per species) and gene inversions (TAP1-TAP2 unit and TAPBP gene) (Shiina et al., 1999; Hosomichi et al., 2006; Chaves et al., 2009; Wang et al., 2012; Ye et al., 2012; Eimes et al., 2013; He et al., 2021). At the same time, information on the structure and location of the MHC-IIA region is virtually lacking for Galliforms (He et al., 2021), although it is thought to be located close to MHC-IIB in other non-passerine lineages, including waterfowl Anseriformes [mallard duck *Anas platyrhynchos* (Ren et al., 2011)], parrots Psittaciformes [kakapo *Strigops habroptila* (Hughes et al., 2008)], storks Ciconiiformes [oriental stork *Ciconia boyciana* (Tsuji et al., 2017)] and ibises Threskiornithidae [crested ibis *Nippon Nippon* (Chen et al., 2015; Lan et al., 2019)]. In comparison to Galliform birds, our knowledge of the architecture of the MHC in other non-passerine birds is even more fragmentary. In general, passerines have much more complex MHC structure because of extensive gene duplications and, on average, they have much higher numbers of MHC gene copies reported than non-passerines (Minias et al., 2018a). For example, a population-wide screening of MHC polymorphism in the sedge warbler *Acrocephalus schoenobaenus* indicated up to 65 different MHC-I alleles per individual (mostly expressed), providing evidence for over 30 duplicated genes (Biedrzycka et al., 2017). A complex MHC gene structure has also been detected in genomic analyses of the zebra finch *Taeniopygia guttata* (Balakrishnan et al., 2010; Ekblom et al., 2011), in which the exact number of gene copies is unclear (Balakrishnan et al., 2010; He et al., 2020)), and the MHC-I and MHC-II genes are not located close together (9.1 cM in the physical arrangement).

Traditional methods used to resolve the MHC architecture in birds included BAC libraries and other plasmids (Guillemot et al., 1988; Kaufman et al., 1999; Moon et al., 2005; Hosomichi et al., 2006; Balakrishnan et al., 2010; Ye et al., 2012; Chen et al., 2015). All these approaches require laborious work, which limited phylogenetic coverage of study taxa and did not allow a broad-scale comparative research on the MHC structure. Thus, information on the architecture of the MHC region was until recently available for only a handful of avian species. However, third-generation sequencing (TGS) with “long-read” technology (reads of over 10 kb) has the potential to alleviate these issues (Van Dijk et al., 2018). Our previous study of TGS-based genomes of birds revealed that long-read based genomes can dramatically improve our knowledge of MHC structure, allowing researchers to reliably quantify gene copy number variation within this region (He et al., 2020). While our previous research primarily focused on the variation in copy numbers of antigen-presenting MHC-I and MHC-II genes, here we elaborate on these results to provide broader information on the gene arrangement patterns within the entire MHC-related region across diverse avian lineages. For this purpose, we studied MHC gene structure in 45 species of birds with available TGS-based genomes (32 non-passerines and 13 passerines). We aimed to 1) explore major evolutionary transitions in MHC structure across birds, and 2) infer the ancestral structure of the MHC in extant basal non-passerine lineages (data complemented with traditional second-generation sequencing genomes). Finally, we retrieved MHC-IIA sequences from TGS-based genomes to quantify the signature of pathogen-driven selection at these genes, as phylogenetically-robust analyses of selection have only been conducted for avian MHC-I exon 3 (Minias et al., 2018b), MHC-IIB exon 2 (Minias et al., 2018b) and exon 3 (Goebel et al., 2017), respectively.

## Materials and Methods

### Compilation of Genomic Data

Publicly available TGS-based genome data (*n* = 45 species) were downloaded from the National Center for Biotechnology Information (NCBI, Bethesda, MD, United States) and the Vertebrate Genomes Project (VGP, vertebrategenomesproject.org; accessed on 8 March 2021). If the same species had independent genome assemblies available in both databases, we focused our analyses on data from NCBI. All GenBank assembly accession numbers, assembly statistics and references are listed in Supplementary Table S1.

To describe the ancestral MHC architecture in birds we complemented the TGS data with traditional “short-read” second-generation sequencing (SGS) genomes available for Palaeognathae and Galloanserae birds. In general, palaeognaths form a monophyletic sister group to all the remaining birds (neognaths) and are considered a basal lineage for extant birds [diverged ca. 100 million years ago, mya (Mitchell et al., 2014; Kuhl et al., 2021)]. Galloanserae (Galliformes and Anseriformes) are a basal group within Neognathae and are thought to have diverged 70–80 mya from Neoaves (Prum et al., 2015; Kuhl et al., 2021). In total, we downloaded and analysed 19 SGS-based Palaeognathae genomes (Supplementary Table S2), as there was only a single TGS-based genome available for this group (emu *Dromaius novaehollandiae*). Within Galloanserae we focused exclusively on waterfowl Anseriformes (13 SGS-based genomes downloaded, Supplementary Table S3), as MHC structure in landfowl (Gallifomes) has already been well characterized [reviewed in (He et al., 2021)]. Data from waterfowl were also used to examine how MHC structure was conserved within a single order.

### Searching for MHC-Related Genes

**FIGURE 1:**
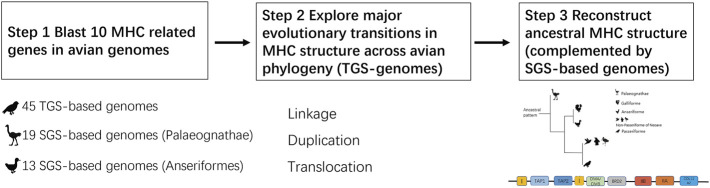
The protocol used to reconstruct the evolution of avian MHC structure.

We selected the key antigen-presenting genes (MHC-I, MHC-IIA and IIB) and several MHC-related genes (TAP1, TAP2, DMA, DMB, TAPBL, BRD2, and COL11A2) for Blast search procedures (Mark et al., 2018) (Figure 1-step1), with e-values < 1e^−5^ and >80% identity over >80% of the length of the query. The last of our targeted genes (COL11A2) was included in the analyses, because it was considered to mark the boundary of the MHC class II region and extended class II region in mammals and ibises (Chen et al., 2015; Shiina et al., 2017). To retrieve contigs with MHC-related genes we implemented algorithms developed and described in our previous study (He et al., 2020). Briefly, BLAST searches were conducted separately for each exon (exon 2, 3 and 4 of MHC-I and MHC-II, other exons excluded because of short length, <100 bp) and based on retrieved data we generated consensus MHC sequences (large proportion of degenerate sites), which were then re-used for Blast searches. Only loci with all three functional major exons (2–4) located within 2 kb of each other were retained. MHC-I and MHC-II sequences with stop-codons were considered pseudogenes [likely to occur in avian genomes because of birth-and-death MHC evolution (Nei et al., 1997)] and excluded from the analyses. All GenBank numbers for our query sequences in Blast (most of them originating from the chicken, and COL11A2 from crested ibis) are listed in Supplementary Table S4. We also confirmed reliability of using these query sequences in passerines (details in Supplementary File 1). As previously shown, TGS-based genomes proved a more accurate data source for predicting the number of MHC loci than SGS-based genomes (He et al., 2020), but TGS-based genomes may also show some variation in assembly quality. Here, in order to check for any possible effects of TGS-based genome quality on our results, we tested for associations between genome contig N50 and predicted MHC gene copy numbers using Spearman correlation.

### Analyses of MHC Architecture

To visualize the structure of the MHC, we extracted regions around the predicted MHC-I or MHC-II genes and plotted them using *gggenes* (https://github.com/wilkox/gggenes), an extension of *ggplot2* package in R (Team, R.D.C., 2018). We only chose regions with MHC-I or MHC-II and associated genes for plotting. To describe phylogenetic distribution of MHC gene arrangement patterns and to explore the linkage patterns within non-passerines (where physical linkage means that genes or gene regions are located within the same contig), we used the complete avian time-calibrated phylogeny by Jetz et al. (Jetz et al., 2012) with a backbone tree by Ericson et al. (Ericson et al., 2006), as available at the BirdTree web server (http://www.birdtree.org). Similar analysis was not feasible for passerines because of a highly complex MHC architecture.

### Selection and Phylogenetic Relationships of MHC-IIA Genes

Selection patterns at the MHC-IIA gene were conducted exclusively for exon 2, which forms the peptide-binding region (α1 domain) of the MHC-II molecule. Available MHC-IIA sequences were extracted from TGS genomes with TBtools (Chen et al., 2020), according to the results from Blast, and translated into amino acid sequences to check functionality. Species with partial MHC-IIA gene sequences were not considered in the analyses. One sequence was randomly selected per species (1–6 sequences available) and we also included a sequence from crested ibis, which was available in GenBank (KP182409). First, the sites under pervasive positive selection (apparent across the whole phylogeny) were identified with Fixed Effects Likelihood (FEL) and Fast Unconstrained Bayesian AppRoximation (FUBAR) models (Kosakovsky Pond and Frost, 2005; Murrell et al., 2013), while the sites under episodic positive selection (apparent at a subset of branches) were recognized using Mixed Effects Model of Evolution (MEME) model (Murrell et al., 2012). All these analyses were run through the Datamonkey webserver (https://www.datamonkey.org/) (Weaver et al., 2018). We considered posterior probabilities >0.90 (FUBAR) or statistical significance *p* < 0.05 (FEL, MEME) as providing enough support for selection signal. Second, the signature of selection was inferred based on the nonsynonymous versus synonymous nucleotide substitution rates (*d*_N_/*d*_S_) in Mega X (Kumar et al., 2018). The *d*_N_/*d*_S_ ratios were calculated across all sites and sites of the peptide binding region, as inferred for humans (Reche and Reinherz, 2003).

## Results

### Retrieving MHC-Related Genes From TGS-Based Genomes

We searched for 10 genes of the MHC family across 45 TGS-based avian genomes and the number of retrieved gene copies varied between species (Tables 1, 2, Supplementary Tables S5, S6), although this variation was unlikely to be driven by differences in genome quality. For highly duplicated genes (>2 copies in at least one species), the number of retrieved copies did not correlate with genome contig N50 values (Spearman correlation: all *p* > 0.05 for MHC class I, IIA, IIB, and COL11A2 in non-passerines). Also, we found no significant association between the number of retrieved MHC-IIA and IIB copies (Spearman: *R* = 0.329, *p* = 0.062).

**TABLE 1:** Gene numbers, linkage, and gene arrangement of MHC-I and MHC-II genes in non-passerine birds (TGS-based genomes).

Species	MHC gene copy number			Gene arrangement and linkage^a^						
	I	IIB	IIA	Type of MHC-I region	Type of MHC-II region	MHC-I and II linkage	MHC-IIA and IIB linkage	MHC-IIA-IIB structure	MHC-IIA and COL11A2 linkage	Linked contig
*Centrocercus minimus*	2	3	1	GalGal-like	GalGal-like	Y	N	—	N	I-related ∼ IIB-related
*Gallus gallus*	2	2	1	GalGal-like	GalGal-like	Y	N	—	N	I-related ∼ IIB-related
*Pavo cristatus*	0	1	1^b^	—	GalGal-like	—	N	—	N	—
*Phasianus colchicus*	2	3	0	GalGal-like	GalGal-like	Y	—	—	N	I-related ∼ IIB-related
*Anas platyrhynchos-2* ^c^	1	1	1	GalGal-like	AnaPla-like	N	Y	IIA ∼ IIB ∼ IIB	Y	IIB-related ∼ IIA
*Anser cygnoides*	6	2	1	GalGal-like	StrHab-like	N	Y	IIA ∼ IIB	Y	IIB-related ∼ IIA
*Cygnus atratus*	2	2	1	GalGal-like	AnaPla-like	N	Y	IIA ∼ IIB ∼ IIB	Y	IIB-related ∼ IIA
*Aythya fuligula*	11	2	1	GalGal-like	AnaPla-like	Y (551 kb)	Y	IIA ∼ IIB ∼ IIB	Y	I-related ∼ IIB-related ∼ IIA
*Cygnus olor*	3	2	1	GalGal-like	AnaPla-like	Y (104 kb)	Y	IIA ∼ IIB ∼ IIB	Y	I-related ∼ IIB-related ∼ IIA
*Streptopelia turtur*	0	0	0	—	—		—	—	—	—
*Phoenicopterus ruber*	9	2	2	GalGal-like-V	NipNip-like (2)^d^	N^e^	Y	(IIA ∼ IIB) × 2	Y	IIB-related ∼ IIA
*Pterocles gutturalis*	5	3	3	GalGal-like	NipNip-like (2)	Y	Y	(IIA ∼ IIB) × 3	N	I-related ∼ IIB-related ∼ IIA
*Tauraco erythrolophus*	4	0	0	GalGal-like	—	—	—	—	N^e^	—
*Calypte anna*	0	0	0	—	—	—	—	—	N^e^	—
*Nyctibius grandis*	5	3	2	GalGal-like	AnaPla-like	Y (972 kb)	Y	IIB ∼ IIA ∼ IIB; IIA ∼ IIB	N	I-related ∼ IIB-related ∼ IIA
*Cuculus canorus*	2	6	1	GalGal-like	AnaPla-like	Y (336 kb)	Y	IIA ∼ IIB ∼ IIB × 5	Y	I-related ∼ IIA ∼ IIB-related
*Alca torda*	4	2	2	GalGal-like-V	NipNip-like (2)	N	Y	(IIA ∼ IIB) × 2	Y	IIB-related ∼ IIA
*Pluvialis apricaria*	3	1	1	GalGal-like	StrHab-like	Y (150 kb)	Y	IIA ∼ IIB	Y	I-related ∼ IIB-related ∼ IIA
*Sterna hirundo*	3	0	1	GalGal-like-V	—	—	—	—	Y	
*Balearica regulorum*	5	2	1	GalGal-like	AnaPla-like	Y	Y	IIA ∼ IIB ∼ IIB	N^e^	I-related ∼ IIB-related ∼ IIA
*Grus nigricollis*	3	2	1	GalGal-like	StrHab-like	N	Y	IIA ∼ IIB	Y	IIB-related ∼ IIA
*Ciconia maguari*	3^d^	2	2	GalGal-like	NipNip-like (2)	N	Y	(IIA ∼ IIB) × 2	N	IIA ∼ IIB, MHC-I-related
*Merops nubicus*	17	3	3	GalGal-like ^f^	NipNip-like (2)^f^	Y^g^	Y	(IIA ∼ IIB) × 2; IIB ∼ IIA	N	I ∼ IIB-related ∼ IIA ∼ I-related
*Falco naumanni*	0	1	1	—	StrHab-like	—	Y	IIA ∼ IIB	N	IIB-related ∼ IIA
*Falco rusticolus*	0	0	0	—	—	—	—	—	N^e^	—
*Picoides pubescens*	5	7	1	—	StrHab-like	Y^e^(228 kb)	Y	IIA ∼ IIB; and others^h^	N	I ∼ IIB-related
										IIB-related ∼ IIA
*Pogoniulus pusillus*	1	4	0	^i^	Only IIB (3)	N	—	Only IIB (3)	N^e^	—
*Melopsittacus undulatus*	3	1	1	—	StrHab-like	Y^e^	Y	IIA ∼ IIB	N	I ∼ IIB-related ∼ IIA
*Strigops habroptila*	2	1	1	GalGal-like	StrHab-like	N	Y	IIA ∼ IIB	N	IIB-related ∼ IIA
*Aquila chrysaetos*	3	3	3	GalGal-like	NipNip-like (3)	N	Y	(IIA ∼ IIB) × 3	Y	IIB-related ∼ IIA
*Bucorvus abyssinicus*	4	8	6	Other^j^	Other^j^	N	Y	Listed in footnote^j^	N	Other^j^
*Cariama cristata*	2	2	2	GalGal-like	NipNip-like (2)	Y (175 kb)	Y	(IIA ∼ IIB) × 2	Y	I-related ∼ IIA ∼ IIB-related

a Note: Linkage means that genes or gene regions were located within the same contig. If the distance between the MHC-I region and IIB-region is more than 100kb, it was provided in the brackets.

b Estimated based on partial exon.

c Genome data ID of AnaPla correspond to GCA_015476345.1 (AnaPla-1) and GCA_900411745.1 (AnaPla-2).

d In NipNip-like, the numbers in brackets suggested duplication numbers of IIA-IIB dyads.

e Although MHC-I and MHC-II genes were not in the same contig, some MHC-I and II related genes were located in the same contig.

f The MHC I-related region contained MHC-II genes (I × 2∼IIB ∼ IIA ∼ TAP1/2 region ∼ I ∼ TAPBP ∼ I × 7) and some MHC-I genes were located in MHC-II region (I × 5∼DMA/B region ∼ BRD2∼IIA ∼ IIB ∼ IIA ∼ IIB).

g CoL11A2 was not adjacent to IIA but in the same contig with BRD2.

h In *Picoides pubescens,* there was one unit of IIA ∼ IIB; and one contig with 6 IIB.

i In *Pogoniulus pusillus*, no TAPs were found in genome data, so we didn’t infer the pattern of MHC-I related region.

j Species-specific gene arrangement was IIA-IIB × 3; (IIB × 2 ∼ I × 2) × 2∼Tap1/2; IIA ∼ IIB ∼ IIA × 3 ∼ BRD2 ∼ DMA/B.

**TABLE 2:** Gene numbers, linkage, and gene arrangement of MHC-I and MHC-II genes in passerine birds (TGS-based genomes).

Species	Number of gene copies				Number of MHC-I and IIB related clusters		Gene linkage (whether in the same contig)			
	TAP1	TAP2	IIA	COL11A2	MHC-I contigs	MHC-IIB contigs	TAP1-TAP2 linkage	MHC-I and TAPs linkage	Existence of MHC-I and IIB linkage	MHC-IIA and IIB linkage
*Acanthisitta chloris*	1	1	1	1	1	1	N	N	N	Y
*Catharus ustulatus*	1	1	1	—	1	2	Y	N	Y (1)^a^	N
*Chiroxiphia lanceolate*	1	1	—	1	2	2	Y	N	Y (1)	—
*Corvus hawaiiensis*	1	1	1	1	4	5	Y	N	N	Y
*Corvus moneduloides*	1	1	—	1	1	1	Y	N	N	—
*Eopsaltria australis*	1	—	1	1	4	24	—	N	Y (3)	N
*Geothlypis trichas*	1	1	1	1	2	3	Y	N	Y (2)	Y
*Hirundo rustica*	1	1	—	1	3	6	Y	N	Y (3)	—
*Manacus vitellinus*	1	1	1	1	20	135	Y	N	Y (5)	N
*Pipra filicauda*	1	1	1	1	13	34	Y	N	Y (2)	N
*Sporophila hypoxantha*	3^b^	1	1	1	1	1	Y^b^	N	N	N
*Sylvia atricapilla*	1	1	—	1	2	2	N	N	N	—
*Taeniopygia guttata*	—	1	—	1	1	3	—	Y (>700 kb)	N	—

a Note:The numbers in brackets suggested the numbers of contigs having both MHC-I and MHC-IIB genes.

b Only one linked TAP1-TAP2 pair found, despite three TAP1 in Sporophila hypoxantha.

In non-passerines, all three antigen-presenting genes showed the highest variation in copy numbers and highest duplication rate: MHC-I (up to 17 copies per species), IIA (up to 6 copies), IIB (up to 8 copies). The copy number of other MHC-related genes was much less variable (up to 2 copies of TAP1, TAP2, DMA, and DMB; a single copy of TAPBP, BRD2, and COL11A2). Most of our targeted genes were successfully retrieved across all study species, except TAPBP, which was only found in Galliformes and 3 other species (*Grus nigricollis, Cariama cristata,* and *Merops nubicus*, Supplementary Table S5).

Among passerines, we found more variation in copy numbers at MHC- I (up to 27 copies) and MHC-IIB (up to 193 copies) when compared with non-passerine birds, and in most species these genes were scattered across multiple contigs (1–20 contigs for MHC-I and 1–135 contigs for MHC-IIB). In contrast, there was only one copy of MHC-IIA retrieved in all study passerines. Duplication of TAP1 was detected in *Sporophila hypoxantha*, but these were linked with only one copy of TAP2 linked with this cluster. There was only one copy of COL11A2 recorded across all studied passerines and it was not adjacent to MHC-IIA gene. We did not successfully retrieve BRD2, TAPBP, DMA and DMB genes in passerines, because the length of BLAST hits was short or there were no hits at all (Supplementary Table S5).

### Describing Reference Gene Arrangement Patterns for Avian MHC

**FIGURE 2:**
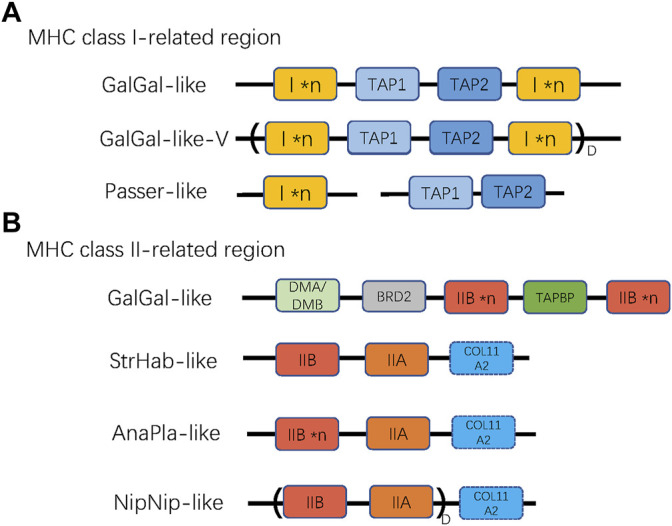
The patterns of gene arrangement in **(A)** MHC class I-related, and **(B)** class II-related regions. *n indicates copy number variation of a single gene (MHC-I or MHC-IIB), while D with parentheses indicates duplication of a linked region. For MHC-I region, GalGal-like-V variant indicates duplicated (I × *n* ∼ TAP1∼TAP2∼I × *n*) unit, and Passer-like pattern indicates lack of linkage between TAPs and MHC-I genes. For MHC-II region, the NipNip-like pattern indicates a duplicated (IIB ∼ IIA) unit. The dashed box of COL11A2 indicates that this gene may be either present or absent in the MHC-II region.

To describe the architecture of the MHC region, we first distinguished two subregions based on the original sequence of the chicken MHC: TAP1, TAP2, and MHC-I (hereafter referred to as “MHC-I-region”) and TAPBP, BRD2, MHC-IIA, and MHC-IIB (hereafter referred to as “MHC-II region”), and identified several reference patterns of gene arrangement based on published BAC sequences (landfowl, mallard, crested ibis, and kakapo) and our own data. For the MHC-I region, we first defined the reference gene arrangement, as recorded in the chicken (henceforth referred to as GalGal-like), where both TAP genes (TAP1 and TAP2) are linked together with MHC-I genes (Figure 2A). Using our data, we also defined another variant of GalGal-like gene arrangement, where the entire MHC-I region (MHC-I × *n* ∼ TAP1∼TAP2∼MHC-I × *n* unit, where *n indicates the number of gene copies) was duplicated (GalGal-like-Variant, henceforth referred to as GalGal-like-V). Finally, we defined a gene arrangement characteristic for passerine birds (henceforth Passerine-like), where MHC-I genes were not linked with TAP1-TAP2 unit (Figure 2A).

Then, we defined four reference gene arrangement patterns for MHC-II region, including: GalGal-like (genes arranged as reported in chickenn; i.e., a duplicated MHC-IIB with one copy of unlinked IIA), AnaPla-like (genes arranged as reported in mallard; one copy of MHC-IIA linked with several copies MHC-IIB), StrHab-like (genes arranged as reported in kakapo; one copy of MHC-IIA linked with one copy of MHC-IIB), and NipNip-like (genes arranged as reported in crested ibis; several copies of MHC-IIA-IIB units). The main differences between these reference patterns were manifested in the linkage and duplication of MHC-IIA and MHC-IIB genes, but also in the position of the COL11A2 gene (Figure 2B).

### MHC Gene Arrangement Patterns Across Avian Phylogeny

**FIGURE 3:**
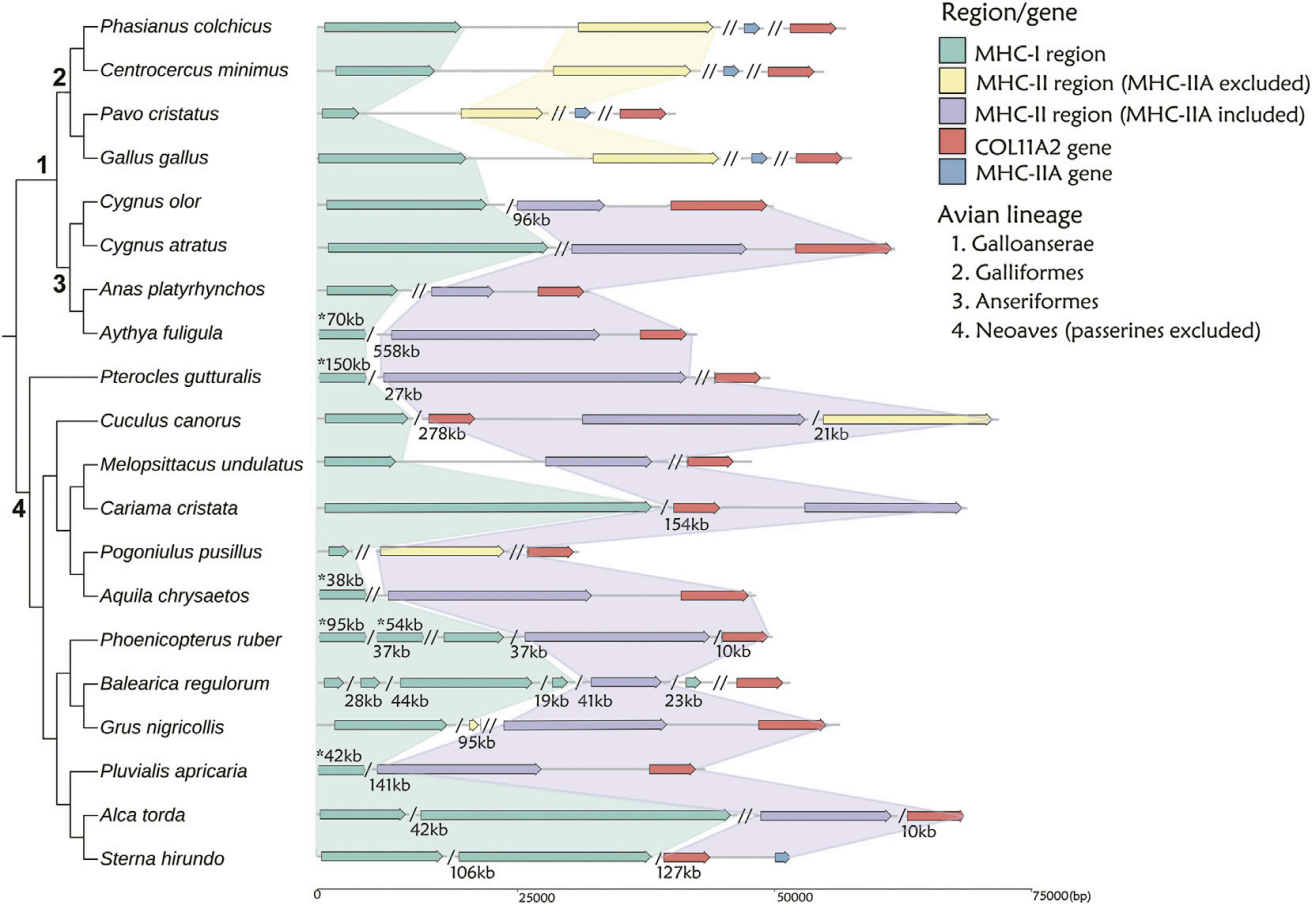
The arrangement of MHC-I and MHC-II regions in 20 non-passerine bird species. The MHC-I- related region is marked with green, while MHC-II region including or excluding MHC-IIA gene is marked with purple and yellow, respectively. MHC-IIA is marked with blue, when not linked to the core MHC-II region. A single slash indicates the same contig or chromosome (numbers listed under the gene arrangement patterns indicate the total length of missing distances associated with single slashes), while a double slash indicates different contig or chromosome. The numbers marked with asterisks (above gene arrangement) indicate the distances that do not match the scale. Note: 1 Visualization of MHC structure for *Anas platyrhynchos* based on genome-2 in Table 1 (GCA_900411745.1), while the distance between MHC-I and MHC-II regions was 310 kb in genome-1 (GCA_015476345.1).

**FIGURE 4:**
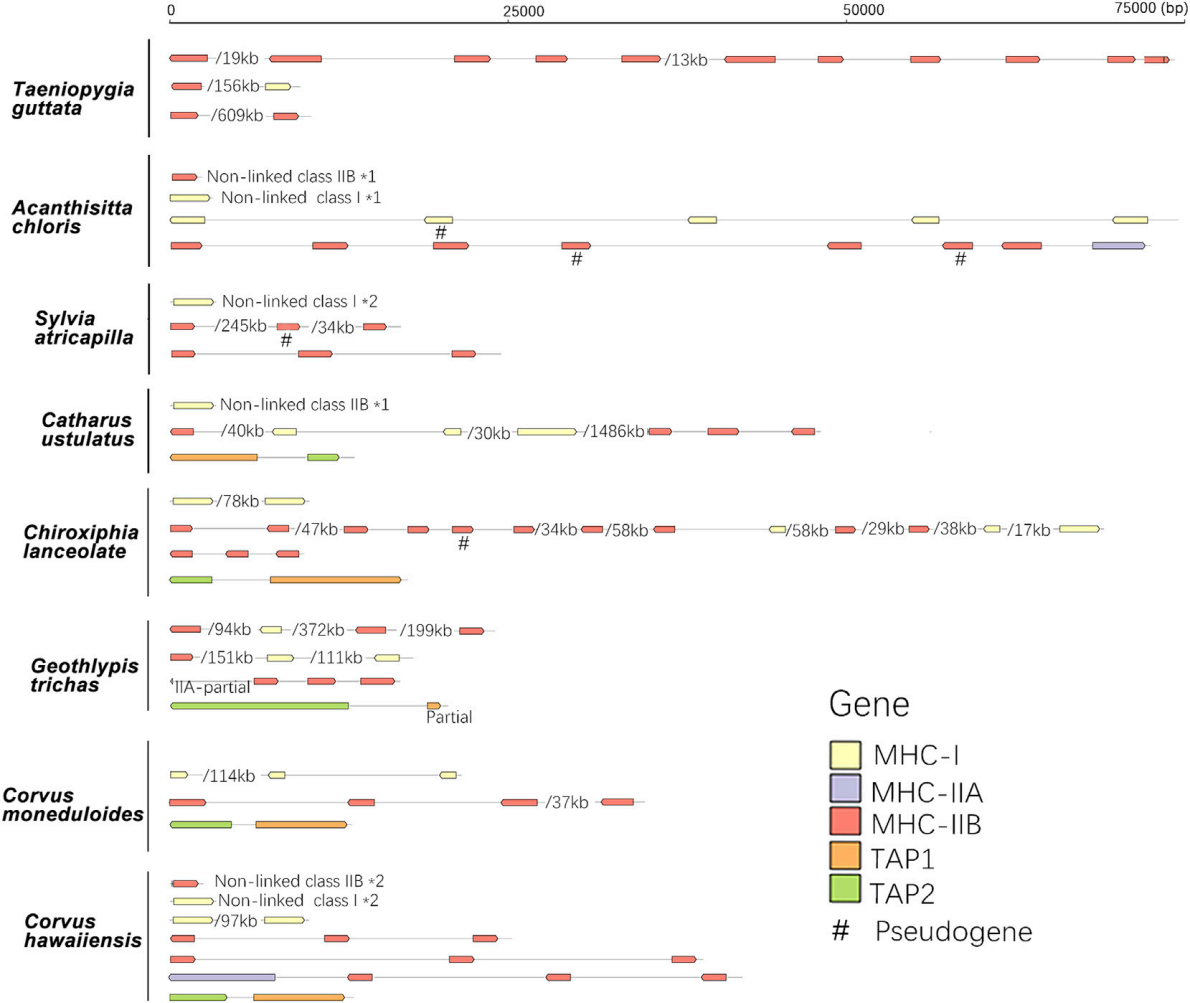
The arrangement of MHC-I, MHC-II, and TAP genes in eight passerine bird species. A single slash indicates the same contig or chromosome and associated numbers indicate the length of missing distances, while *number indicates the number of duplication events. A single slash indicates the same contig or chromosome (numbers listed under the gene arrangement patterns indicate the total length of missing distances associated with single slashes).

We used TGS-based genomes to examine the MHC gene arrangement in twenty non-passerine (Figure 3) and eight passerine (Figure 4) species. Other species were excluded from the analyses because their MHC-related regions were more fragmented across different contigs (see Supplementary Figure S1 and Supplementary Table S5 for details).

First, in gallonanseres (Galliformes and Anseriformes) we found three distinct patterns of MHC gene arrangement. In Galliformes, the MHC-I and MHC-II regions were compact and tightly clustered into a single MHC core region (11.12–14.17 kb distance between MHC-I and MHC-II; 23.92–42.14 kb total length). The MHC-IIA and COL11A2 genes were found on separated chromosomes or scaffolds, and we found no evidence for their close physical linkage with the MHC core region. In contrast, MHC-IIA and IIB genes were closely linked in Anseriformes, including only one MHC-IIA gene and a variable number (2–4) of MHC-IIB gene copies. This was consistent with AnaPla-like (IA-IIB-IIB pattern, *n* = 4 species) or StrHab-like (IIA-IIB pattern, *n* = 1 species) gene arrangements. At the same time, there was no apparent close physical linkage between MHC-I and MHC-II regions in Anseriformes, which were located within 100 kb from each other, and perhaps even on separate scaffolds/chromosomes (Table 1; Figure 3). The MHC-I and MHC-IIB copy numbers in *Anas platyrhynchos* were 3 and 7 (respectively) in the first genome (AnaPla-1), while only one gene copy per class was detected in the second genome (AnaPla-2) (Table 3, details in Supplementary Table S5). In contrast, previous BAC-based studies of the mallard revealed five MHC-I and five MHC-IIB gene copies (Moon et al., 2005; Ren et al., 2011). The discrepancy may reflect insufficient quality (coverage) of available genome assemblies or it might also be related to haplotype variation between individuals; however it should not affect the analysis of MHC genes arrangement patterns.

**TABLE 3:** Gene numbers, linkage, and gene arrangement of MHC-I and MHC-II genes in waterfowl Anseriformes (SGS- and TGS-based genomes) TGS-based data were marked with asterisks (*).

Species	Family	MHC gene copy number			Gene linkage		
		I^a^	IIA	IIB^a^	I-IIB related region	IIA-IIB	IIA-COL11A2
*Anser cygnoides* *	Anatidae	6	1	2	N	Y	Y
*Anas platyrhynchos-2* *	Anatidae	1	1	1	Y (24,085 kb)^b^	Y	Y
*Anas zonorhyncha*	Anatidae	8	1	4	Y (103 kb)	Y	Y
*Anser brachyrhynchus*	Anatidae	2	1	0 (0, 0, 0)	N	—	Y
*Anser indicus*	Anatidae	1	1	1	N	Y	N
*Anseranas semipalmata*	Anseranatidae	0 (1, 1, 1)	1	0 (1, 1, 2)	N	N	Y
*Asarcornis scutulata*	Anatidae	2	1	1	N	Y	Y
*Aythya fuligula* *	Anatidae	11	1	3	Y (558.7 kb)	Y	Y
*Branta canadensis*	Anatidae	2	1	0 (0, 1, 1)	N	N	Y
*Cairina moschata*	Anatidae	1	1	0 (1, 0, 0)	Y (73.6 kb)	Y	Y
*Chauna torquata*	Anhimidae	0 (0, 2, 1)	—	0 (0, 1, 1)	N	—	—
*Cygnus atratus* *	Anatidae	2	1	2	N	Y	Y
*Cygnus cygnus*	Anatidae	1	1	0	N	—	Y
*Cygnus olor* *	Anatidae	2	1	2	Y (59.5 kb)	Y	Y
*Heteronetta atricapilla*	Anatidae	1	1	0	N	—	N
*Nettapus auritus*	Anatidae	1	1	1	N	N	Y
*Oxyura jamaicensis*	Anatidae	1	1	0 (0, 1, 1)	N	N	N
*Stictonetta naevosa*	Anatidae	1	1	1	N	N	Y

a Note: If exons 2-4 were not found within a single conting, the number of hits containing separate exons 2, 3, and 4 was listed in the brackets.

b The numbers in the brackets indicate the distance between the two regions.

In other non-passerine birds, duplication and translocation patterns of MHC-related genes were more complex (Figure 3). First, the size of both MHC-I and II-related regions expanded to a varying degree (in comparison to Galliformes), and there was little evidence for a tight linkage between these regions, except for two species from the orders of Pterocliformes (27 kb distance between MHC-I and MHC-II in *Pterocles gutturalis*) and Psittaciformes (26 kb distance in *Melopsittacus undulates*). Second, a close linkage between MHC-IIA/IIB and COL11A2 was conserved in several non-passerine lineages (IIA-IIB-COL11A2 pattern, *n* = 7 species), although in two species we recorded gene inversion within this region (*Cuculus canorus* and *Sterna hirundo*). Also, approximately half of our study non-passerine species (*n* = 12) showed evidence of CoL11A2 translocation, as it was not linked with MHC-II region. Third, we found evidence of MHC-II gene duplication in eight species (2-3 duplicated copies) and both MHC-IIA and IIB genes were always duplicated as a single unit (NipNip-like pattern; Table 1). Finally, we found unique MHC gene arrangement patterns in two species, where either MHC-IIA-IIB genes were translocated into the MHC-I region (I × 2∼IIB ∼ IIA ∼ TAP1/2 region ∼ I in *Merops nubicus*) or MHC-IIA (but not MHC-IIB) gene showed evidence of duplication (IIA ∼ IIB ∼ IIA × 3 in *Bucorvus abyssinicus*).

When compared with non-passerines, the most notable features of passerine MHC included translocations of TAP genes, genomic expansion (MHC-related genes found across different chromosomes), and extensive duplication of MHC-I and MHC-IIB genes (Figure 4). In 12 of 13 species, TAP1 and TAP2 genes showed no apparent linkage with MHC-I genes (found in different contigs), while in one species (*Taeniopygia guttata*) we found TAPs and MHC-I within a single contig, but the distance between them was >700 kb (Table 2). At the same time, both TAP genes were tightly linked in all study passerines (<10 kb distance in 8 species). In most passerines we retrieved a single copy of MHC-IIA gene, but found no evidence for its linkage with MHC-IIB. Some passerine lineages retained a linkage between MHC-I and MHC-II (we retrieved many contigs containing both MHC-I and IIB genes), but the patterns of gene arrangement showed large inter-specific variation and we found no evidence for linkage between these regions in some species (Table 2 and Supplementary File 2).

### Reconstructing Ancestral MHC Architecture Complemented by SGS-Based Genomes (Palaeognathae and Anseriformes)

To complement our analyses of TGS-based genomes, we also examined the MHC gene arrangement in the ancestral avian lineages (Palaeognathae and Anseriformes) using SGS-based data. Within Palaeognathae, 13 species had poor resolution of the MHC region (MHC-related genes scattered across more than three contigs or lacking some key genes, e.g., MHC-IIA), so they were not included in the analyses (Supplementary Table S6). In the remaining seven species, the MHC core region was compact and small in size (<100 kb) (Supplementary Table S5, Supplementary Figure S2). In all these species, MHC-related contigs also included a linked MHC-IIA-IIB unit, but gene arrangement showed variation between different Palaeognathae lineages, including three reference patterns of StrHab-like (Apterygiformes, Casuariiformes, Rheiformes, and Tinamiformes), AnaPla-like (Apterygiformes, Struthioniformes, and Tinamiformes), and NipNip-like (Tinamiformes).

In contrast, our analysis of SGS-based data in Anseriformes showed that the pattern of MHC structure was highly conserved within this lineage, including the GalGal-like class I-related region and the AnaPla-like class II-related region, but with no tight linkage between them. Three SGS-based Anseriform genomes (*Anas zonorhyncha, Asarcornis scutulata*, and *Cairina moschata*) showed exactly the same pattern of MHC gene arrangement, as previously found in Anseriformes based on TGS data (Table 3). Another four species did not have IIA-IIB clusters, but IIA was tightly linked with COL11A2 (Table 3). Phylogenetic analysis indicated a presence of two separate MHC clusters within Anatidae family, including *Anser*, *Branta*, and *Cygnus* genera in one clade (red in Supplementary Figure S3) and all other anatids in the second one (blue in Supplementary Figure S3). The patterns of MHC gene arrangement were inconsistent with phylogenetic relationships (Supplementary Figure S3), suggesting that the lack of the MHC-IIA-IIB cluster could be an artefact resulting from low resolution of SGS-based genomes.

### Selection at MHC-IIA Genes in Birds

The MHC-IIA gene showed no evidence of duplication across most avian lineages and most of the retrieved nucleotide MHC-IIA sequences (incomplete in some species) could be translated into functional amino acid sequences (except for BucAbu-IIA2, PteGut-IIA1 and PhoRub-IIA1). In taxa with duplicated MHC-IIA (2–6 gene copies, *n* = 9 species), the amino acid sequences were highly conserved and showed polymorphism in six species.

We found evidence of weak positive (diversifying) selection at MHC-IIA across avian phylogeny. The *d*_N_/*d*_S_ ratio was 0.37 for the entire α1 domain (exon 2) and it was similar between the peptide-binding region (PBR, as identified based on human HLA structure (Reche and Reinherz, 2003)) and non-PBR (0.458 and 0.339, respectively). Only 1–2 sites were identified as under pervasive positive selection (FEL and FUBAR), but many more sites (*n* = 11) showed signature of episodic positive selection (MEME). Only one site (position 81, Supplementary Figure S4) was found as under positive selection using all three approaches (Supplementary Figure S4). Four positively selected sites at the avian MHC-IIA (as identified in this analysis) corresponded to human PBR and seven human PBR sites were negatively selected in birds.

## Discussion

**FIGURE 5:**
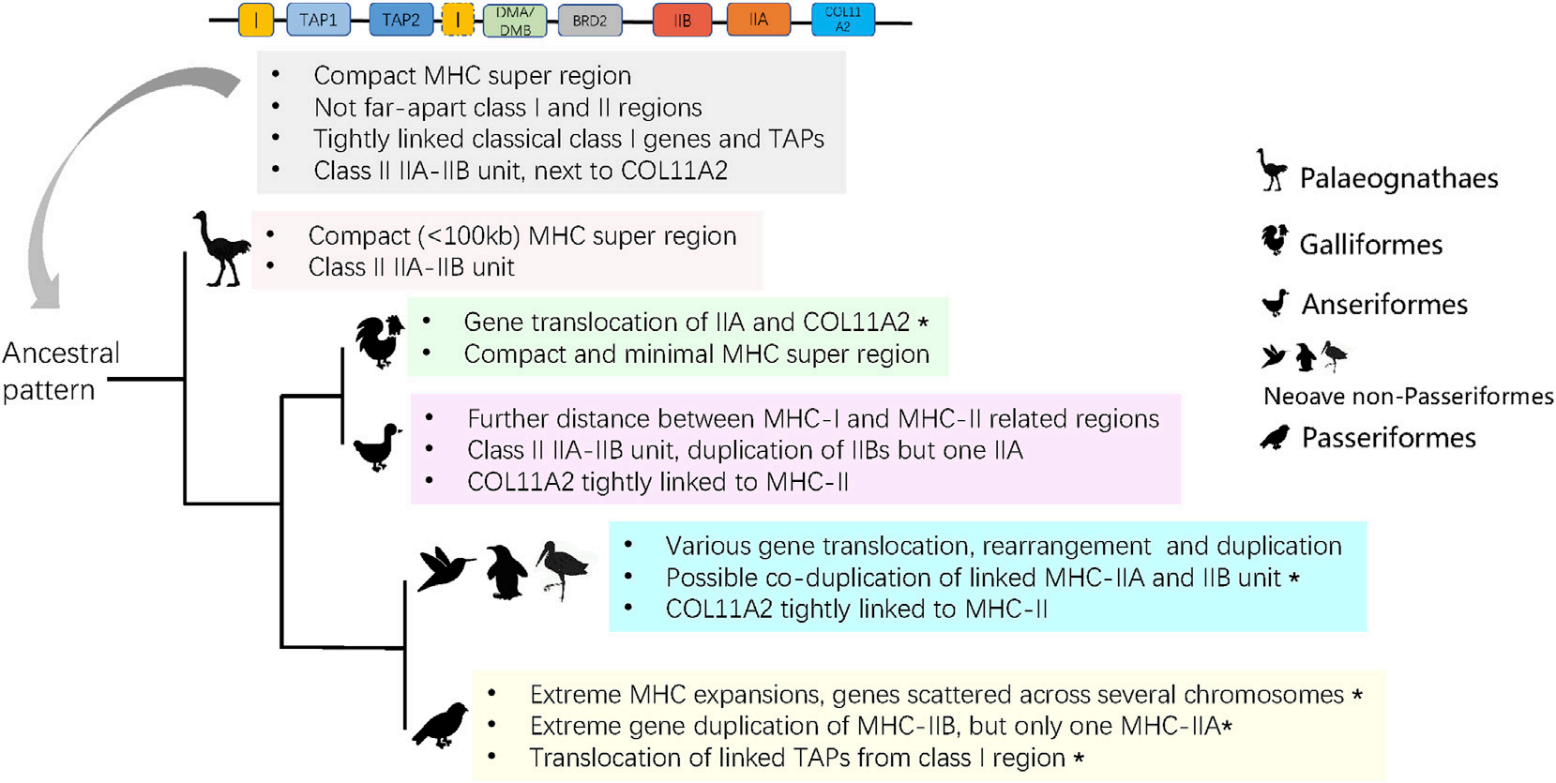
The predicted ancient MHC architecture in birds and its evolution in derived groups. Asterisks indicate characters that are unique to a group.

In this study, we attempted to reconstruct the origin and evolutionary history of avian MHC architecture using high-quality TGS-based genomes. An analysis of MHC architecture in Palaeognathae and Galloanserae indicated that the ancestral avian MHC appeared to have linked MHC-I and MHC-II regions, in which the MHC-I region contained antigen processing genes (TAPs), and the MHC-II region contained both IIA and IIB units (not found in Galliformes) (Figure 5). We also found several evolutionary changes in the core MHC architecture. First, the minimal MHC (<100 kb) was only detected in Galliformes and Palaeognathae; in other birds the MHC region was >100 kb in length, and in Passeriformes it was scattered across different chromosomes. Second, most avian lineages had tightly linked IIA and IIB genes within the MHC-II region, although several structure variants could be distinguished based on gene duplication patterns. Finally, our analyses provided support for strong purifying selection acting on MHC-IIA genes.

### Ancestral Structure and Major Evolutionary Events in Avian MHC Evolution

To date, the architecture of the MHC in birds has been poorly known outside of Galliformes. Most studies on avian MHC have reported polymorphisms at MHC-I or MHC-IIB genes using high-through sequencing (Biedrzycka et al., 2017; Minias et al., 2019; Qin et al., 2021), but did not examine arrangement of MHC genes on a chromosomal scale. We found that 1) the ancestral avian MHC was compact in size (<100 kb), class I and II regions were separated by DM (DMA and DMB) genes, 2) MHC-I genes were tightly linked with TAPs, and 3) the MHC-II region contained linked IIA and IIB genes (Figure 5).

In humans, the entire MHC is larger and more complex than the typical avian MHC, with all three main regions linked into a single class I-class III-class II structure, which seems to be conserved in eutherian mammals (Shiina et al., 2017; Abduriyim et al., 2019). In contrast, the ancestral avian MHC is more similar to the MHC architecture found in marsupials [e.g., opossum *Monodelphis domestica* (Gouin et al., 2006)] and reptiles, such as tuatara (Miller et al., 2015), and crocodiles (Jaratlerdsiri et al., 2014a), as all of these lineages have an adjacent MHC-I and II regions that are not seperated by MHC-III. In fact, genomic inversion might be responsible for the formation of the typical MHC arrangement in placental mammals (Kaufman, 2018). In our analyses, we found MHC-I and II regions within single contigs in most avian groups, but they were separated in Galliformes (MHC-IIA unlinked with IIB). During evolution, some groups of birds experienced different degrees of translocation and various gene rearrangements or duplications (Figure 5), and we observed several unique restructuring events, including translocation of MHC-IIA and COL11A2 in Galliformes, a single MHC-IIA gene linked with duplicated IIB genes in Anseriformes, co-duplication of MHC-IIA-IIB unit in Neoave non-passerines, and highly variable patterns of gene duplication in Passeriformes (Figure 5). In particular, passerines showed an extreme gene expansion, extreme variation in MHC-IIB copy number associated with only one MHC-IIA copy, and translocation of TAPs (Figure 5). These patterns may be associated with the rapid diversification of passerines during the Paleogene period.

In mammals, the MHC-II region often contains a linked IIA-IIB unit, although the number and organization of these genes may largely differ between lineages. For example, felines lack MHC-DQ genes, while there was an expansion in the number of DR genes in the cow *Bos taurus* and DP genes were replaced with DI/DY genes in the sheep *Ovis aries* (Trowsdale and Knight, 2013). At the same time, studies in cetaceans have found inversion and translocation events resulting in the separation of the MHC-IIA and IIB sub-regions (De Sá et al., 2019). In birds, pioneering research on MHC architecture was conducted on the domestic chicken, providing evidence for a translocation of MHC-IIA from the core MHC-II region, resulting in the loss of close physical linkage between MHC-IIA and IIB genes (Salomonsen et al., 2003). Further analyses of other landfowl taxa indicated that this translocation event was prevalent across the entire clade of Galliformes and, thus, it probably occurred prior to the divergence of this lineage from Anseriformes [ca. 77 mya according to (Prum et al., 2015)]. Previously, the linked MHC-IIA-IIB unit has been detected in BAC-based MHC research on several non-galliform birds (Chen et al., 2015; Tsuji et al., 2017), but our study is the first to demonstrate its occurrence across many diverse avian lineages. We found three combinations of MHC-IIA and IIB gene arrangement (Figure 2), suggesting duplications of either MHC-IIB gene alone or duplications of MHC-IIA and IIB genes as a single unit. An apparent duplication of MHC-IIB (without MHC-IIA) was found in Anseriformes (Table 3), and high number of MHC-IIB genes associated with a single IIA gene were also recorded in Passeriformes (Table 2). This pattern has likely evolved independently in both clades, as it was not recorded in lineages directly ancestral to passerines. In fact, in most lineages of Neaoves (except Passeriformes) we observed a simultaneous co-duplication of MHC-IIA and IIB genes. Previously, two ancient MHC-IIB lineages were suggested to appear by a duplication event before the radiation of birds (ca. 100 mya), and the clades of Galloanserae and Passeriformes were found to have retained different gene lineages (Goebel et al., 2017), which also supports our finding on the independent MHC-II evolution in these two clades.

Besides the antigen presenting MHC-I and II genes, the MHC region contains genes that are required for antigen processing such as TAP1, TAP2, and TAPBP. TAP genes (TAP1 and TAP2) are located within the MHC-II region in mammals (Trowsdale and Knight, 2013), but in saltwater crocodile *Crocodylus porosus* they were found within the MHC-I region (Jaratlerdsiri et al., 2014a), which is generally consistent with what we observe in birds. In our study, all TAP genes in non-passerine species were located within the MHC-I region, but tightly linked TAPs translocated from MHC-I in Passeriformes, which appears to be unique for this order (Figure 5). We also observed duplication of TAPs in four divergent avian species (*Pavo cristatus, Alca torda, Sterna hirundo*, and *Phoenicopterus ruber*), and the same pattern was previously found in reptiles [saltwater crocodile (Jaratlerdsiri et al., 2014a)] and marsupials [tammar wallaby *Notamacropus eugenii* (Siddle et al., 2011)]. We also observed variation in the organization of COL11A2 gene, which marks a boundary of MHC-II region in birds [e.g., crested ibis (Chen et al., 2015; Lan et al., 2019)] and is located within an extended MHC-II region in mammals (Shiina et al., 2017). We found that in most avian lineages COL11A2 gene was located within the MHC-II region, but in Galliformes and Passeriformes it was not in obvious physical linkage with MHC-II. Location of other MHC-related genes, such as DMA, DMB, and BRD2 (details in Supplementary Table S4), was consistent with the architecture of ancestral avian MHC model, showing no major rearrangement events during bird evolution.

### Purifying Selection in Avian MHC-IIA Genes

Both MHC-IIA and IIB genes code for different peptide-binding subunits of a single MHC-II molecule, but the signature of pathogen-driven selection may differ between these two domains. Strong positive (diversifying) selection has often been reported at MHC-IIB exon 2 (Minias et al., 2018b), but MHC-IIA genes were clearly under-researched in terms of selection patterns. Analyses of MHC-IIA in reptiles [crocodiles (Jaratlerdsiri et al., 2014b)] provided evidence for low polymorphism and purifying selection at these genes. Also, allelic diversity at MHC-IIB was estimated to be 10 times higher than at MHC-IIA in DR region, and five times higher in DQ and DP regions in humans (James et al., 2015). However, other comparative analyses of MHC polymorphism in both mammals and fish indicated that allelic richness at MHC-IIA genes may be similar to or even exceed the level of polymorphism observed at MHC-IIB, and that MHC-IIA may be subject to strong positive selection (Cíková et al., 2011; Bracamonte et al., 2015).

Since most of the birds have only one copy of IIA gene, a single α subunit is expected to associate with multiple β subunits (coded by IIB genes) to form diverse MHC-II molecules. Consequently, polymorphism of the MHC-IIA gene may be of vital adaptive importance for birds. Nevertheless, we are aware of only two population-wide screening studies of MHC-IIA genes in birds (chicken and the Leach’s storm-petrel *Oceanodroma leucorhoa*), and both showed a low level of polymorphism. Specifically, there were only four nucleotide and one amino acid polymorphisms detected within a single MHC-IIA gene in the chicken (Salomonsen et al., 2003), whereas two IIA genes were found in the storm-petrel: DAA with a single allele and DBA with three alleles differing from each other by single non-synonymous substitutions (Rand et al., 2019). This clearly contrasted with markedly diverged MHC-IIB alleles, as found in the same study (Rand et al., 2019). Here, we only examined MHC-IIA sequences from one individual per species, so we had no insight into the intra-specific MHC-IIA variation and we can only discuss polymorphism across species. Despite the fact that we observed some order-specific MHC-IIA amino acid sequences (Supplementary Figure S4), more than 1/3 of sites at PBR exons were found to be negatively selected and our analyses of *d*_N_/*d*_S_ ratios also supported a strong signature of negative (purifying) selection. This seems to support the hypothesis that most polymorphism of the avian MHC-II concentrates within the β domain (MHC-IIB) of this molecule. We failed to detect MHC-IIA sequences in some species with our Blast searches. This most likely reflects insufficient genome coverage, particularly near centromere or telomere regions which contain DNA sequence repeats and are difficult to sequence. Further improvements in sequencing and assembly methodology may in the future improve resolution of the avian MHC-IIA region.

## Conclusion

Over the last decade, improvements in high-throughput sequencing have greatly expanded our knowledge of MHC diversity within population, particularly in non-model vertebrate species. However, short-read sequencing has limited ability to reveal the genomic structure of the MHC region. Here, we took advantage of long-read-based genomic data to provide physical maps of the MHC architecture across the bird phylogeny, revealing the ancestral avian MHC structure and major rearrangement events in the evolution of this region. We hope that our results will provide a valuable template for future comparative analyses of avian MHC, allowing more effective tests for macroevolutionary associations of MHC polymorphism with life history, ecology, or pathogen exposure in birds. Currently, our study confirms that long-read sequencing technology has a great potential to advance our understanding of MHC structure and provide novel insights into the mechanisms that drive MHC evolution. At the same time, we acknowledge that future improvements in genome assembly, particularly in resolving haplotypes (Cheng et al., 2021) may provide much better resolution of the MHC region and its extraordinary complexity across vertebrates (e.g., *via* identification of copy number variation between haplotypes).

## Data Availability

The original contributions presented in the study are included in the article/Supplementary Material, further inquiries can be directed to the corresponding authors.

## References

[B1] Abduriyim S. Zou D. H. Zhao H. (2019). Origin and Evolution of the Major Histocompatibility Complex Class I Region in Eutherian Mammals. Ecol. Evol. 9, 7861–7874. 10.1002/ece3.5373 31346446 PMC6636196

[B2] Alcaide M. Liu M. Edwards S. V. (2013). Major Histocompatibility Complex Class I Evolution in Songbirds: Universal Primers, Rapid Evolution and Base Compositional Shifts in Exon 3. PeerJ 1, e86. 10.7717/peerj.86 23781408 PMC3685324

[B3] Balakrishnan C. N. Ekblom R. Völker M. Westerdahl H. Godinez R. Kotkiewicz H. (2010). Gene Duplication and Fragmentation in the Zebra Finch Major Histocompatibility Complex. BMC Biol. 8, 2929. 10.1186/1741-7007-8-29 PMC2907588 20359332

[B4] Biedrzycka A. O’Connor E. Sebastian A. Migalska M. Radwan J. Zając T. (2017). Extreme MHC Class I Diversity in the Sedge Warbler (*Acrocephalus Schoenobaenus*); Selection Patterns and Allelic Divergence Suggest that Different Genes Have Different Functions. BMC Evol. Biol. 17, 159. 10.1186/s12862-017-0997-9 28679358 PMC5497381

[B5] Blum J. S. Wearsch P. A. Cresswell P. (2013). Pathways of Antigen Processing. Annu. Rev. Immunol. 31, 443–473. 10.1146/annurev-immunol-032712-095910 23298205 PMC4026165

[B6] Bracamonte S. E. Baltazar-Soares M. Eizaguirre C. (2015). Characterization of MHC Class II Genes in the Critically Endangered European Eel (*Anguilla anguilla*). Conservation Genet. Resour. 7, 859–870. 10.1007/s12686-015-0501-z

[B7] Chaves L. D. Krueth S. B. Reed K. M. (2009). Defining the turkey MHC: Sequence and Genes of the B Locus. J. Immunol. 183, 6530–6537. 10.4049/jimmunol.0901310 19864609

[B8] Chen C. Chen H. Zhang Y. Thomas H. R. Frank M. H. He Y. (2020). TBtools: An Integrative Toolkit Developed for Interactive Analyses of Big Biological Data. Mol. Plant 13, 1194–1202. 32585190 10.1016/j.molp.2020.06.009

[B9] Chen L.-C. Lan H. Sun L. Deng Y.-L. Tang K.-Y. Wan Q.-H. (2015). Genomic Organization of the Crested Ibis MHC Provides New Insight into Ancestral Avian MHC Structure. Sci. Rep. 5, 7963. 10.1038/srep07963 25608659 PMC4302302

[B10] Cheng H. Concepcion G. T. Feng X. Zhang H. Li H. (2021). Haplotype-resolved De Novo Assembly Using Phased Assembly Graphs with Hifiasm. Nat. Methods 18, 170–175. 10.1038/s41592-020-01056-5 33526886 PMC7961889

[B11] Cíková D. Bellocq J. Baird S. Piálek J. Bryja J. (2011). Genetic Structure and Contrasting Selection Pattern at Two Major Histocompatibility Complex Genes in Wild House Mouse Populations. Heredity 106, 727–740. 10.1038/hdy.2010.112 20823902 PMC3186229

[B12] Eimes J. A. Reed K. M. Mendoza K. M. Bollmer J. L. Whittingham L. A. Bateson Z. W. (2013). Greater Prairie Chickens Have a Compact MHC-B with a Single Class IA Locus. Immunogenetics 65, 133–144. 10.1007/s00251-012-0664-7 23179555

[B13] Ekblom R. Stapley J. Ball A. D. Birkhead T. Burke T. Slate J. (2011). Genetic Mapping of the Major Histocompatibility Complex in the Zebra Finch (*Taeniopygia guttata*). Immunogenetics 63, 523–530. 10.1007/s00251-011-0525-9 21494955

[B14] Ericson P. G. P. Anderson C. L. Britton T. Elzanowski A. Johansson U. S. Källersjö M. (2006). Diversification of Neoaves: Integration of Molecular Sequence Data and Fossils. Biol. Lett. 2, 543–547. 10.1098/rsbl.2006.0523 17148284 PMC1834003

[B15] Goebel J. Promerová M. Bonadonna F. Mccoy K. D. Serbielle C. Strandh M. (2017). 100 Million Years of Multigene Family Evolution: Origin and Evolution of the Avian MHC Class IIB. BMC Genomics 18, 460. 10.1186/s12864-017-3839-7 28610613 PMC5470263

[B16] Gouin N. Deakin J. E. Miska K. B. Miller R. D. Kammerer C. M. Graves J. A. M. (2006). Linkage Mapping and Physical Localization of the Major Histocompatibility Complex Region of the *Marsupial Monodelphis* Domestica. Cytogenet. Genome Res. 112, 277–285. 10.1159/000089882 16484784

[B17] Guillemot F. Billault A. Pourquié O. Béhar G. Chaussé A. M. Zoorob R. (1988). A Molecular Map of the Chicken Major Histocompatibility Complex: the Class II Beta Genes Are Closely Linked to the Class I Genes and the Nucleolar Organizer. EMBO J. 7, 2775–2785. 10.1002/j.1460-2075.1988.tb03132.x 3141149 PMC457068

[B18] He C. Zhao L. Xiao L. Xu K. Ding J. Zhou H. (2021). Chromosome Level Assembly Reveals a Unique Immune Gene Organization and Signatures of Evolution in the Common Pheasant. Mol. Ecol. Resour. 21, 897–911. 10.1111/1755-0998.13296 33188724

[B19] He K. Minias P. Dunn P. O. (2020). Long-Read Genome Assemblies Reveal Extraordinary Variation in the Number and Structure of MHC Loci in Birds. Genome Biol. Evol. 13, evaa270. 10.1093/gbe/evaa270 PMC7875000 33367721

[B20] Hosomichi K. Shiina T. Suzuki S. Tanaka M. Shimizu S. Iwamoto S. (2006). The Major Histocompatibility Complex (Mhc) Class IIB Region Has Greater Genomic Structural Flexibility and Diversity in the Quail Than the Chicken. BMC genomics 7, 322. 10.1186/1471-2164-7-322 17184537 PMC1769493

[B21] Hughes C. R. Miles S. Walbroehl J. M. (2008). Support for the Minimal Essential MHC Hypothesis: a Parrot with a Single, Highly Polymorphic MHC Class II B Gene. Immunogenetics 60, 219–231. 10.1007/s00251-008-0287-1 18431567

[B22] James R. Halliwell J. A. Hayhurst J. D. Paul F. Peter P. Steven g. e., M. (2015). The IPD and IMGT/HLA Database: Allele Variant Databases. Nuclc Acids Res. 43, D423. 10.1093/nar/gku1161 PMC4383959 25414341

[B23] Jaratlerdsiri W. Deakin J. Godinez R. M. Shan X. Peterson D. G. Marthey S. (2014a). Comparative Genome Analyses Reveal Distinct Structure in the Saltwater Crocodile MHC. PLoS One 9, e114631. 10.1371/journal.pone.0114631 25503521 PMC4263668

[B24] Jaratlerdsiri W. Isberg S. R. Higgins D. P. Miles L. G. Gongora J. (2014b). Selection and Trans-species Polymorphism of Major Histocompatibility Complex Class II Genes in the Order Crocodylia. Plos One 9, e87534. 10.1371/journal.pone.0087534 24503938 PMC3913596

[B25] Jetz W. Thomas G. H. Joy J. B. Hartmann K. Mooers A. O. (2012). The Global Diversity of Birds in Space and Time. Nature 491, 444–448. 10.1038/nature11631 23123857

[B26] Kaufman J. (2018). Generalists and Specialists: A New View of How MHC Class I Molecules Fight Infectious Pathogens. Trends Immunol. 39, 367–379. 10.1016/j.it.2018.01.001 29396014 PMC5929564

[B27] Kaufman J. Milne S. Göbel T. W. F. Walker B. A. Jacob J. P. Auffray C. (1999). The Chicken B Locus Is a Minimal Essential Major Histocompatibility Complex. Nature 401, 923–925. 10.1038/44856 10553909

[B28] Kosakovsky Pond S. L. Frost S. D. W. (2005). Not so Different after All: a Comparison of Methods for Detecting Amino Acid Sites under Selection. Mol. Biol. Evol. 22, 1208–1222. 10.1093/molbev/msi105 15703242

[B29] Kuhl H. Frankl-Vilches C. Bakker A. Mayr G. Nikolaus G. Boerno S. T. (2021). An Unbiased Molecular Approach Using 3′-UTRs Resolves the Avian Family-Level Tree of Life. Mol. Biol. Evol. 38, 108–127. 10.1093/molbev/msaa191 32781465 PMC7783168

[B30] Kumar S. Stecher G. Li M. Knyaz C. Tamura K. (2018). MEGA X: Molecular Evolutionary Genetics Analysis across Computing Platforms. Mol. Biol. Evol. 35, 1547–1549. 10.1093/molbev/msy096 29722887 PMC5967553

[B31] Lan H. Zhou T. Wan Q. H. Fang S. G. (2019). Genetic Diversity and Differentiation at Structurally Varying MHC Haplotypes and Microsatellites in Bottlenecked Populations of Endangered Crested Ibis. Cells 8, 377. 10.3390/cells8040377 PMC6523929 31027280

[B32] Lankat-Buttgereit B. Tampé R. (1999). The Transporter Associated with Antigen Processing TAP: Structure and Function. FEBS Lett. 464, 108–112. 10.1016/s0014-5793(99)01676-2 10618487

[B33] Mark J. Irena Z. Yan R. Yuri M. Scott M. G. Madden T. L. (2018). NCBI BLAST: a Better Web Interface. Nucleic Acids Res. 36, W5–W9. 10.1093/nar/gkn20 PMC2447716 18440982

[B34] Miller H. C. O’Meally D. Ezaz T. Amemiya C. Marshall-Graves J. A. Edwards S. (2015). Major Histocompatibility Complex Genes Map to Two Chromosomes in an Evolutionarily Ancient Reptile, the Tuatara *Sphenodon punctatus* . G3 (Bethesda) 5, 1439–1451. 10.1534/g3.115.017467 25953959 PMC4502378

[B35] Minias P. Pikus E. Anderwald D. (2019). Allelic Diversity and Selection at the MHC Class I and Class II in a Bottlenecked Bird of Prey, the White-tailed Eagle. BMC Evol. Biol. 19, 2–13. 10.1186/s12862-018-1338-3 30611206 PMC6321662

[B36] Minias P. Pikus E. Whittingham L. A. Dunn P. O. (2018a). Evolution of Copy Number at the MHC Varies across the Avian Tree of Life. Genome Biol. Evol. 11, 17–28. 10.1093/gbe/evy253 PMC6319602 30476037

[B37] Minias P. Pikus E. Whittingham L. A. Dunn P. O. (2018b). A Global Analysis of Selection at the Avian MHC. Evolution 72, 1278–1293. 10.1111/evo.13490 29665025

[B38] Mitchell K. J. Llamas B. Soubrier J. Rawlence N. J. Worthy T. H. Wood J. (2014). Ancient DNA Reveals Elephant Birds and Kiwi Are Sister Taxa and Clarifies Ratite Bird Evolution. Science 344, 898–900. 10.1126/science.1251981 24855267

[B39] Moon D. A. Veniamin S. M. Parks-Dely J. A. Magor K. E. (2005). The MHC of the Duck (*Anas platyrhynchos*) Contains Five Differentially Expressed Class I Genes. J. Immunol. 175, 6702–6712. 10.4049/jimmunol.175.10.6702 16272326

[B40] Murrell B. Moola S. Mabona A. Weighill T. Sheward D. Kosakovsky Pond S. L. (2013). FUBAR: a Fast, Unconstrained Bayesian Approximation for Inferring Selection. Mol. Biol. Evol. 30, 1196–1205. 10.1093/molbev/mst030 23420840 PMC3670733

[B41] Murrell B. Wertheim J. O. Moola S. Weighill T. Scheffler K. Kosakovsky Pond S. L. (2012). Detecting Individual Sites Subject to Episodic Diversifying Selection. Plos Genet. 8, e1002764. 10.1371/journal.pgen.1002764 22807683 PMC3395634

[B42] Nei M. Gu X. Sitnikova T. (1997). Evolution by the Birth-And-Death Process in Multigene Families of the Vertebrate Immune System. Proc. Natl. Acad. Sci. 94, 7799–7806. 10.1073/pnas.94.15.7799 9223266 PMC33709

[B43] O'connor E. A. Westerdahl H. Burri R. Edwards S. V. (2019). Avian MHC Evolution in the Era of Genomics: Phase 1.0. Cells 8, 1152. 10.3390/cells8101152 PMC6829271 31561531

[B44] O'connor E. A. Westerdahl H. (2021). Tradeoffs in Expressed Major Histocompatibility Complex Diversity Seen on a Macro‐evolutionary Scale Among Songbirds. Evolution 75, 1061–1069. 10.1111/evo.14207 33666228

[B45] Prum R. O. Berv J. S. Dornburg A. Field D. J. Townsend J. P. Lemmon E. M. (2015). A Comprehensive Phylogeny of Birds (Aves) Using Targeted Next-Generation DNA Sequencing. Nature 526, 569–573. 10.1038/nature15697 26444237

[B46] Qin S. D. Dunn P. O. Yang Y. Liu H. Y. He K. (2021). Polymorphism and Varying Selection within the MHC Class I of Four *Anas* Species. Immunogenetics 73, 395. 10.1007/s00251-021-01222-9 34195858

[B47] Rand L. M. Woodward C. May R. Ackerman R. A. Tweedie B. Zicarelli T. B. (2019). Divergence between Genes but Limited Allelic Polymorphism in Two MHC Class II A Genes in Leach's Storm-Petrels *Oceanodroma Leucorhoa* . Immunogenetics 71, 561–573. 10.1007/s00251-019-01130-z 31506710 PMC7050443

[B48] Reche P. A. Reinherz E. L. (2003). Sequence Variability Analysis of Human Class I and Class II MHC Molecules: Functional and Structural Correlates of Amino Acid Polymorphisms. J. Mol. Biol. 331, 623–641. 10.1016/s0022-2836(03)00750-2 12899833

[B49] Ren L. Yang Z. Wang T. Sun Y. Guo Y. Zhang Z. (2011). Characterization of the MHC Class II α-chain Gene in Ducks. Immunogenetics 63, 667–678. 10.1007/s00251-011-0545-5 21660591

[B50] Sá A. L. A. d. Breaux B. Burlamaqui T. C. T. Deiss T. C. Sena L. Criscitiello M. F. (2019). The Marine Mammal Class II Major Histocompatibility Complex Organization. Front. Immunol. 10, 696. 10.3389/fimmu.2019.00696 31019512 PMC6459222

[B51] Salomonsen J. Marston D. Avila D. Bumstead N. Johansson B. Juul-Madsen H. (2003). The Properties of the Single Chicken MHC Classical Class II ? Chain (B-LA) Gene Indicate an Ancient Origin for the DR/E-like Isotype of Class II Molecules. Immunogenetics 55, 605–614. 10.1007/s00251-003-0620-7 14608490

[B52] Shiina T. Blancher A. Inoko H. Kulski J. K. (2017). Comparative Genomics of the Human, Macaque and Mouse Major Histocompatibility Complex. Immunology 150, 127–138. 10.1111/imm.12624 27395034 PMC5214800

[B53] Shiina T. Shimizu C. Oka A. Teraoka Y. Imanishi T. Gojobori T. (1999). Gene Organization of the Quail Major Histocompatibility Complex ( MhcCoja) Class I Gene Region. Immunogenetics 49, 384–394. 10.1007/s002510050511 10199914

[B54] Siddle H. V. Deakin J. E. Coggill P. Wilming L. G. Harrow J. Kaufman J. (2011). The Tammar Wallaby Major Histocompatibility Complex Shows Evidence of Past Genomic Instability. BMC Genomics 12, 421. 10.1186/1471-2164-12-421 21854592 PMC3179965

[B55] Team, R.D.C. (2018). R: A Language and Environment for Statistical Computing. R Found. Stat. Comput,. Available at: https://www.R-project.org

[B56] Ting J. P. Trowsdale J. (2002). Genetic Control of MHC Class II Expression. Cell 109 (Suppl. l), S21–S33. 10.1016/s0092-8674(02)00696-7 11983150

[B57] Trowsdale J. Knight J. C. (2013). Major Histocompatibility Complex Genomics and Human Disease. Annu. Rev. Genom. Hum. Genet. 14, 301–323. 10.1146/annurev-genom-091212-153455 PMC4426292 23875801

[B58] Tsuji H. Taniguchi Y. Ishizuka S. Matsuda H. Yamada T. Naito K. (2017). Structure and Polymorphisms of the Major Histocompatibility Complex in the Oriental Stork, Ciconia Boyciana. Sci. Rep. 7, 42864. 10.1038/srep42864 28211522 PMC5314415

[B59] Van Dijk E. L. Jaszczyszyn Y. Naquin D. Thermes C. (2018). The Third Revolution in Sequencing Technology. Trends. GENETICS 34, 666–681. 10.1016/j.tig.2018.05.008 29941292

[B60] Wang B. Ekblom R. Strand T. M. Portela-Bens S. Höglund J. (2012). Sequencing of the Core MHC Region of Black Grouse (Tetrao Tetrix) and Comparative Genomics of the Galliform MHC. BMC Genomics 13, 553. 10.1186/1471-2164-13-553 23066932 PMC3500228

[B61] Weaver S. Shank S. D. Spielman S. J. Li M. Muse S. V. Kosakovsky Pond S. L. (2018). Datamonkey 2.0: A Modern Web Application for Characterizing Selective and Other Evolutionary Processes. Mol. Biol. Evol. 35, 773–777. 10.1093/molbev/msx335 29301006 PMC5850112

[B62] Whittingham L. A. Dunn P. O. Freeman-Gallant C. R. Taff C. C. Johnson J. A. (2018). Major Histocompatibility Complex Variation and Blood Parasites in Resident and Migratory Populations of the Common Yellowthroat. J. Evol. Biol. 31, 1544–1557. 10.1111/jeb.13349 29964353

[B63] Ye Q. He K. Wu S.-Y. Wan Q.-H. (2012). Isolation of a 97-kb Minimal Essential MHC B Locus from a New reverse-4D BAC Library of the golden Pheasant. PloS one 7, e32154. 10.1371/journal.pone.0032154 22403630 PMC3293878

[B64] Zeng Q.-Q. He K. Sun D.-D. Ma M.-Y. Ge Y.-F. Fang S.-G. (2016). Balancing Selection and Recombination as Evolutionary Forces Caused Population Genetic Variations in golden Pheasant MHC Class I Genes. BMC Evol. Biol. 16, 42. 10.1186/s12862-016-0609-0 26892934 PMC4758006

