# Supplementary material for: Reconstructing Macroevolutionary Patterns in Avian MHC Architecture With Genomic Data
Source: Front Genet. 2022 Feb 17;13:823686. doi: 10.3389/fgene.2022.823686 (PMC8893315; doi:10.3389/fgene.2022.823686)
Supplement: Supplementary file 1 [file DataSheet1.pdf]

Suppl Figure 1 The arrangement of MHC class I and II-related regions in 33 non-passerines. A single slash indicates the same contig or chromosome (numbers listed under the gene arrangement patterns indicate the total length of missing distances associated with single slashes), while a double slash indicates different contig or chromosome. The numbers marked with asterisks (above gene arrangement) indicate the distances that do not match the scale.

Suppl Figure 2 The arrangement of MHC class I and II-related regions and COL11A2 in Palaeognathae. *Anomalopteryx didiformis* can't be find in BirdTree web server, so we listed it separately. The genes with stars indicated them were not complete in our Blast results.

Suppl Figure 3 Patterns of MHC class I and II-related regions gene arrangement in MHC class I-related and class II-related regions in Anseriformes.

Suppl Figure 4 The alignment of represented amino acid sequences predicted in 32 studied birds. The dots indicate sequences with the same amino acid, and a dashed line represents the gap. A 25% threshold in Geneious generated the consensus sequences. One sequence is chosen to represent the species when the species has more than 1 IIA sequence. Results of three methods (MEME, FEL and FUBAR) were represented in order. PSS, positively selected site; NSS, negatively selected site.

Suppl file 1 The verification of the availability of using our listed queries (MHC-related genes from non-passerine) in Passerines' Blast.

Suppl file 2 Excepting these core class I-related, IIB-related, and IIA-related regions listed in Table 1 (manuscript), there were also some linked genes in the non-passerines genome (Table Suppl 2).

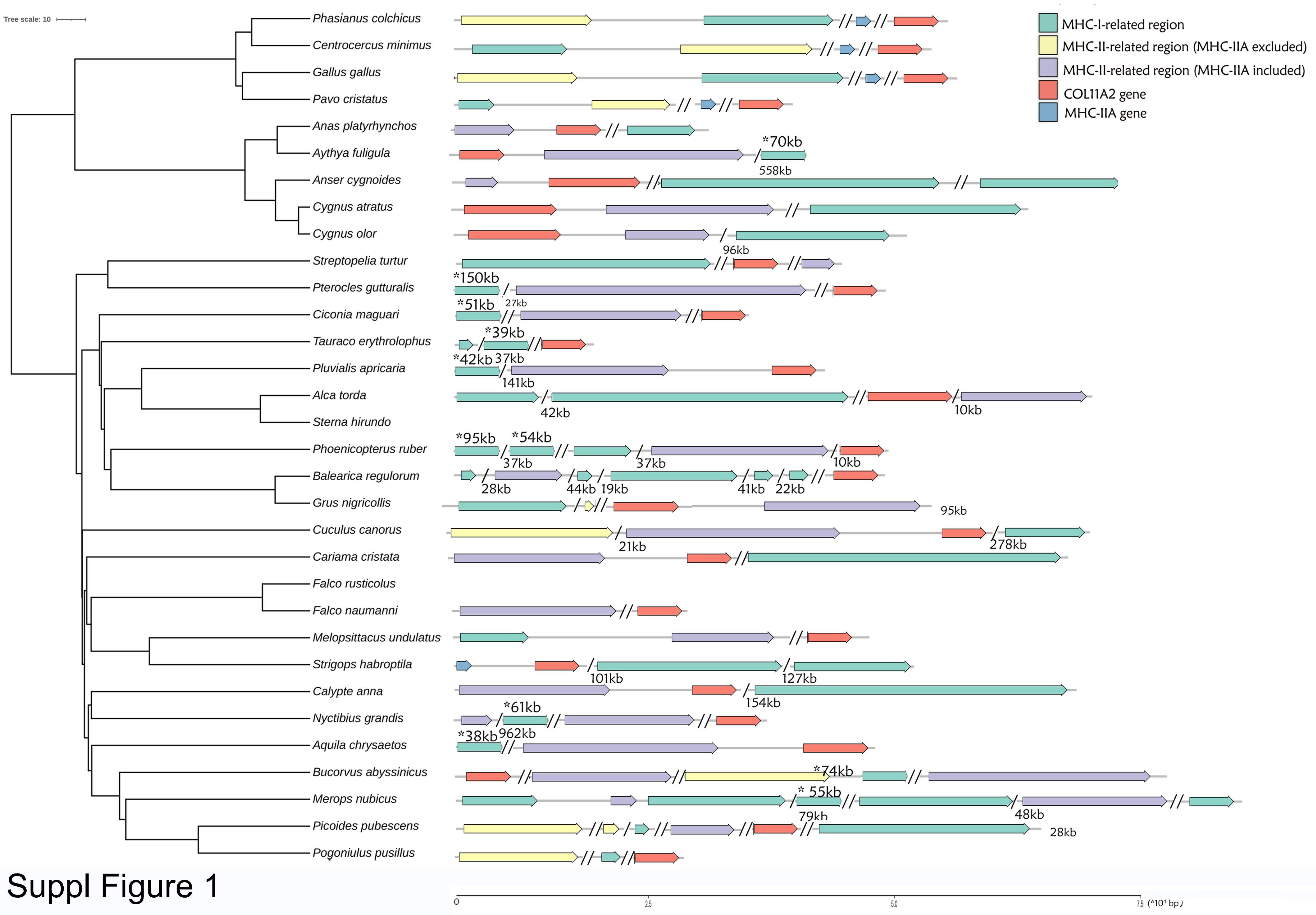

Suppl Figure 1

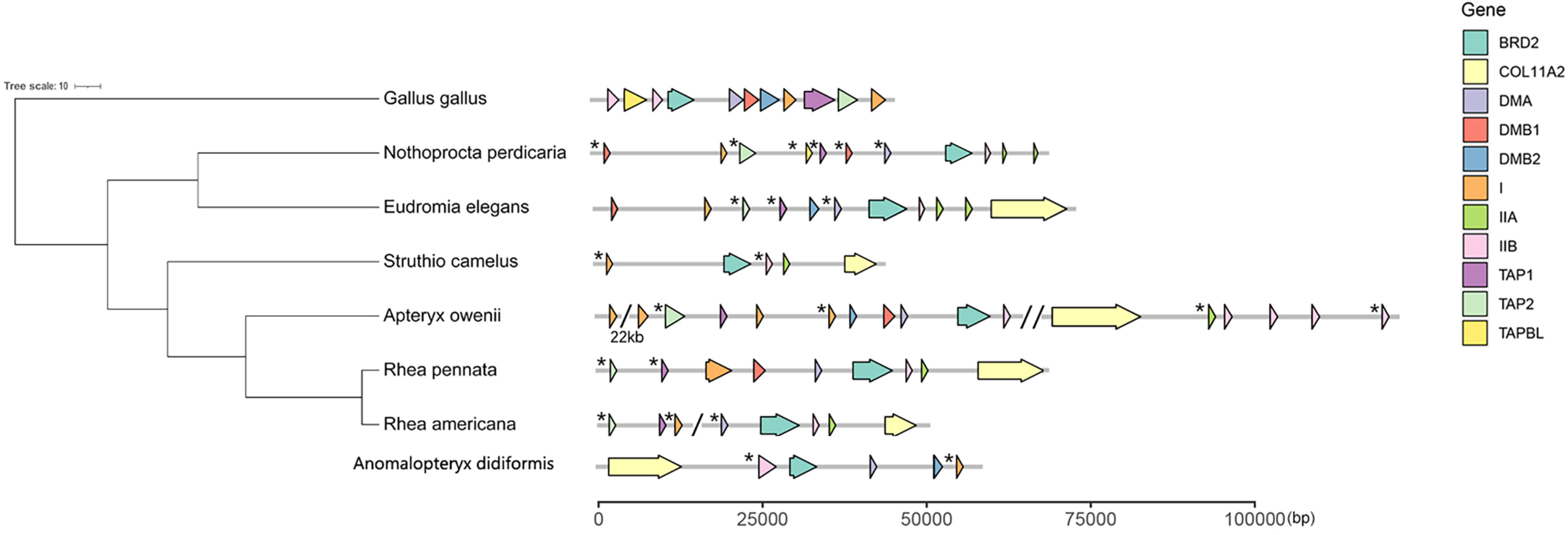

Suppl Figure 2

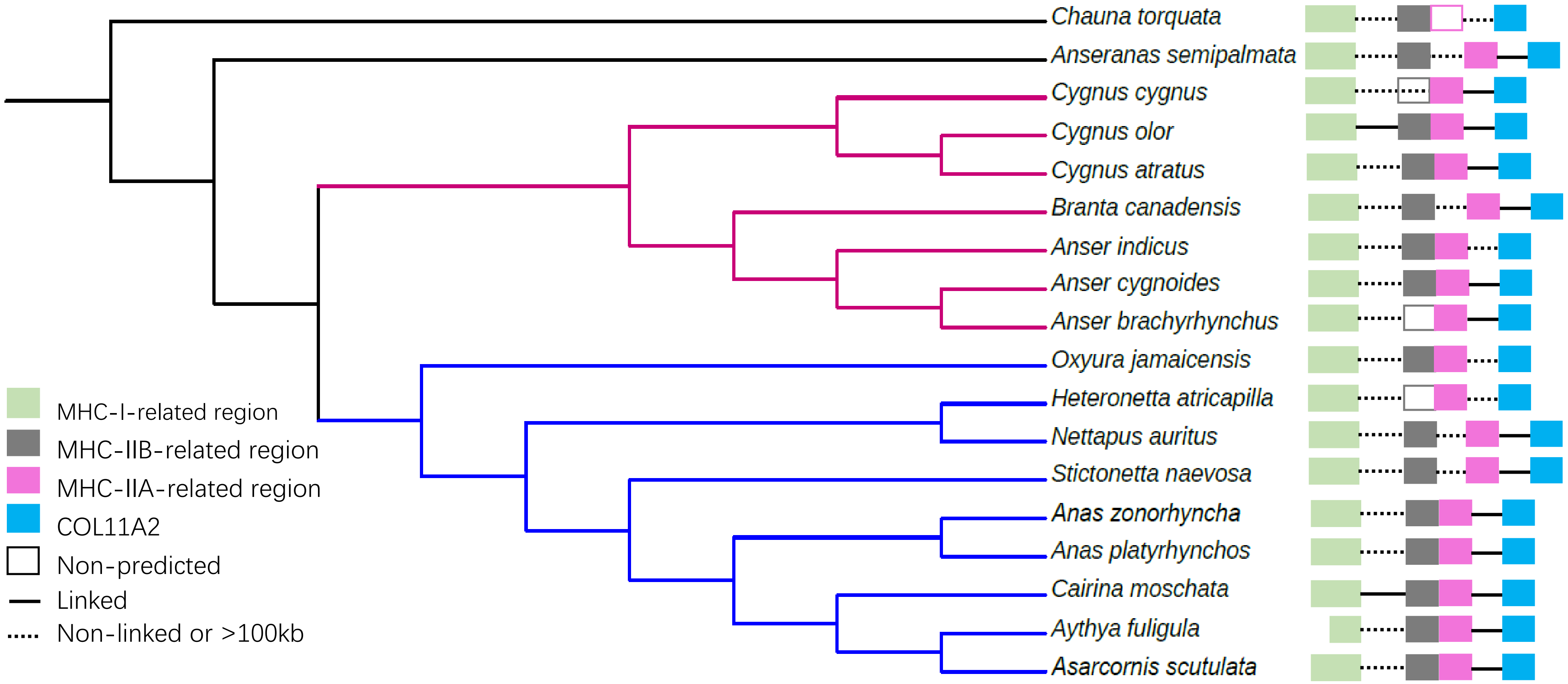

Suppl Figure 3

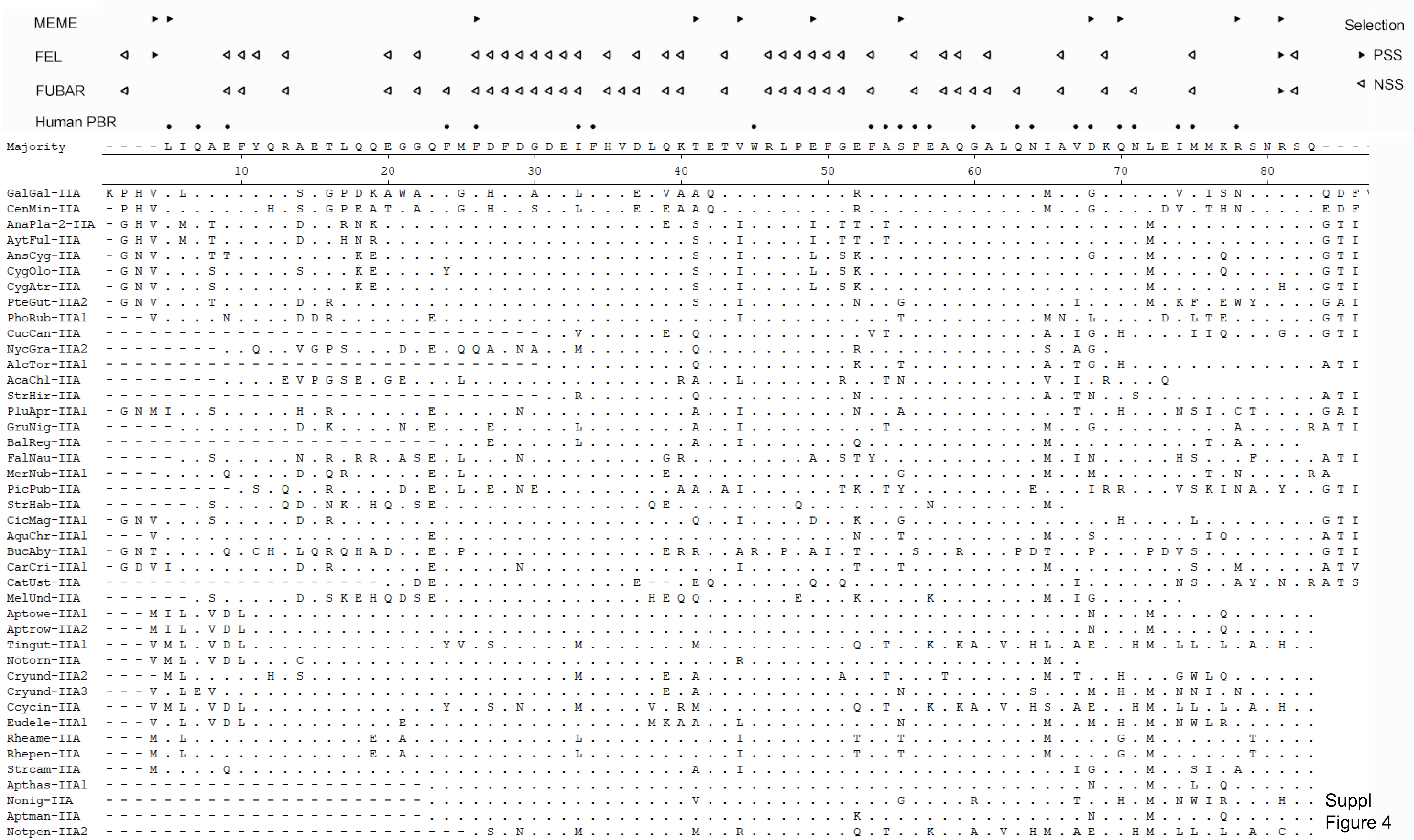

Suppl Figure 4

Suppl file 1

The verification of the availability of using our listed queries (MHC-related genes from non-passerine) in Passerines' Blast

Except for the class I and IIB genes, the other MHC-related genes (TAP1, TAP2, DMB, TAPBL, and IIA) were tested using reported BACs containing MHC-related genes in zebra finch. We Blasted these BACs with our queries, and for other genes, the queries were listed in Table S2), and revealed a perfect match between the genes reported in 6 published MHC BACs and our estimated. This proved that our MHC-related queries set can be used in Passerine.

Table Suppl 1 The detailed information about Blast results in zebra finch's BACs

BAC clone (GenBank)	Reported MHC genes	Blast result	Hits	Similarity (%)	Length of the query (bp)	Start location	End location	E-value
TGAA-323J16 (AC191651.2)	IIB	IIB	IIB-exon4	80.488	82	24572	24653	7.66E-17
			IIB-exon4	83	100	27797	27698	5.51E-25
			IIB-exon3	84.559	272	28542	28277	7.21E-86
			IIB-exon4	81	100	72598	72499	2.85E-22
			IIB-exon3	83.824	272	73343	73078	3.73E-83
			IIB-exon3	81.56	282	81963	82234	5.19E-75
			IIB-exon2	82.449	245	103459	103703	8.10E-72
			IIB-exon3	89.046	283	104342	104618	5.91E-106
			IIB-exon4	87.85	107	105223	105329	3.25E-34
TGAA-351E14 (AC191861.2)	IIB	IIB	IIB-exon3	89.474	95	4815	4909	3.48E-33
			IIB-exon2	82.449	245	25514	25758	8.60E-72
			IIB-exon3	89.007	282	26469	26744	1.80E-106
			IIB-exon4	85.981	107	27329	27435	1.79E-31
			IIB-exon4	81.013	79	129450	129528	2.84E-16
			IIB-exon4	82	100	133043	132944	7.12E-24
			IIB-exon3	82.721	272	133788	133523	2.50E-79
TGAA_047O03 (AC192431.2)	Unsure	IIB	IIB-exon3	83.63	281	18323	18601	7.89E-85
			IIB-exon3	81.55	271	115031	114767	1.63E-74
			IIB-exon2	71.233	219	115714	115496	4.59E-30
TGAA-157B03 (AC192433.2)	TAPBL, IIB	TAPBL,IIB	TAPBL	69.78	182	57151	56985	3.97E-13
			TAPBL	65.207	434	57526	57116	7.18E-10
			TAPBL	71.429	112	58396	58285	7.18E-10
			TAPBL	72.289	83	68304	68222	4.53E-06
			TAPBL	69.091	165	68675	68527	3.05E-08
			TAPBL	71.053	114	68773	68884	3.72E-07
			IIB-exon3	83.63	281	98474	98196	8.20E-85
			IIB-exon2	72.603	219	117019	117237	2.16E-34
			IIB-exon3	81.181	271	117711	117975	7.19E-73
TGAC-167E04/ TGAC-86122 (AC232854.2)	TAP1,TAP2	TAP1,TAP2	TAP2	73.214	168	161	327	3.23E-20
			TAPBL*	67.67	631	46612	46022	6.19E-30
			TAPBL	68.404	307	47207	46920	9.81E-15

			TAP1	68.075	213	49807	50019	4.74E-16
			TAP1	67.55	302	50886	51181	9.15E-19
			TAP1	69.6	125	51200	51324	5.41E-09
			TAP1	75.581	86	51781	51866	1.27E-10
	Class I	Class I, DMB	I-exon2	78.707	263	5596	5858	6.64E-79
			I-exon3	83.15	273	6341	6613	1.98E-79
			I-exon3	86.594	276	6341	6613	1.26E-94
TGAC-102M22 (AC232985.2)			I-exon4	90.11	273	6961	7233	2.75E-109
			DMB1	83.333	48	21801	21754	1.33E-06
			DMB1	71.304	115	21838	21733	3.82E-07
			TAPBL*	66.81	232	60716	60941	8.16E-09

Note: * The TAPBL gene has several exons, and only 1-2 hits were not considered as a real one.

Suppl file 2

Excepting these core class I-related, IIB-related, and IIA-related regions listed in Table 1 (manuscript), there were also some linked genes in the non-passerines genome (Table Suppl 2).

Table Suppl 2 The complement information about linked contig in non-passerines

Species	Other contigs/ Note
Pavo cristatus	Duplicated TAP1~2; TAPBP was not with other MHC related genes
Anas platyrhynchos-1 [1]	Duplicated class I-related region, of which one was adjunct with IIB genes
Anas platyrhynchosa-2 [1]	Class I region with DMA, DMB, and BRD2
Anser cygnoides	Class I region contains DMA and DMB; BRD2 is separated; duplicated class I-related region (in different chromosomes).
Cygnus atratus	Class I region contains DMA and DMB
Cuculus canorus	Class I and II related regions are re-order
Sterna hirundo	IIA~COL11A2; BRD2~DMA/DMB
Grus nigricollis	TAPBP was not with other MHC related genes
Merops nubicus	I*2
Picoides pubescens	IIB*2; I*4
Pogoniulus pusillus	IIB*3
Cariama cristata	Class I and II related regions are re-order
